# Effect of a Mindfulness Training Program on the Impulsivity and Aggression Levels of Adolescents with Behavioral Problems in the Classroom

**DOI:** 10.3389/fpsyg.2016.01385

**Published:** 2016-09-22

**Authors:** Clemente Franco, Alberto Amutio, Luís López-González, Xavier Oriol, Cristina Martínez-Taboada

**Affiliations:** ^1^Department of Psychology, Faculty of Educational Sciences, University of AlmeríaAlmería, Spain; ^2^Department of Social Psychology and Methodology of the Behavioral Sciences, Faculty of Psychology, University of the Basque CountrySan Sebastian, Spain; ^3^Institute of Educational Sciences, University of BarcelonaBarcelona, Spain; ^4^Department of Management and Public Policies, Universidad de Santiago de ChileSantiago, Chile

**Keywords:** mindfulness, impulsivity, aggressiveness, school failure, students

## Abstract

**Objective:** The aim of the present study was to analyze the effects of a mindfulness training psycho-educative program on impulsivity and aggression levels in a sample of high school students.

**Methods:** A randomized controlled trial with pre-test–post-test measurements was applied to an experimental group and a control group (waiting list). The Barratt Impulsivity Scale (BIS-11) Patton et al. (1995) and the Aggression Questionnaire (Buss and Perry, 1992) were used.

**Results:** Statistical analyses showed a significant decrease in the levels of impulsivity and aggressiveness in the experimental group compared with the control group. These results have important implications for improving the level of academic engagement and self-efficacy of students and for reducing school failure.

**Conclusion:** This is one of the first studies showing the effectiveness of mindfulness training at reducing impulsive and aggressive behaviors in the classroom. The efficacy of mindfulness-based programs is emphasized.

## Introduction

Aggressive behavior in both children and adolescents is considered a complex phenomenon that involves multiple factors and manifests in a variety of forms. Among the variables related to this phenomenon are personal traits (e.g., psychopathy, neuroticism, impulsivity, search for sensations), socio-emotional (lack of empathy, self-esteem, personal values), and cognitive variables (e.g., maladaptive schemas and dysfunctional thoughts) (Condon et al., 2013; Estévez et al., 2016; Orue et al., 2016; Pellerone et al., 2016).

Diverse sources and causes of impulsivity and aggressiveness in students are discussed in the literature, including parental style and insecure attachment, peer pressure (Estévez et al., 2016), conflictual relations with the teachers (Settanni et al., 2015), lack of emotional self-control, especially of negative affectivity (Peters et al., 2015; Sanger and Dorjee, 2015), emotional avoidance (Mestre et al., 2012), low compassion (Morley et al., 2016), and even neuroanatomical (Thijssen et al., 2015), among others. Students with aggressive behavior show deficiency in emotional self-control and empathy, features pertaining to the emotional intelligence trait. Therefore, these students will face more difficulties to deal with social situations and their incapacity to adequately managing their emotions may lead them to behave in aggressive ways before uncertain situations (Mestre et al., 2012; Inglés et al., 2014).

Impulsivity is a risk factor associated with reactive aggression and antisocial behavior during adolescence (Orue et al., 2016). In particular, impulsive motor behavior appears to be the factor that seems to discriminate better between aggressive and non-aggressive adolescents (Oberle et al., 2011; Andreu et al., 2013). Thus, impulsive adolescents without sufficient emotional control and no ability to delay gratification are driven by the emotional momentum and little or inadequate forethought. Furthermore, impulsivity and aggressiveness are related, on the one hand, to maladaptive or risk-taking behaviors such as substance abuse or sexual promiscuity (Paydary et al., 2016) and, on the other hand, to mental disorders, attention-deficit hyperactivity disorder, reading problems, and poor academic results (Fix and Fix, 2013; Nelson et al., 2015).

School related tasks require the ability to use and regulate emotions in order to increase concentration, develop intrinsic motivation, and control impulsive thinking and hostility. Adolescents with high levels of aggression use non-productive coping strategies to a greater extent, whereas less aggressive adolescents mainly focus on strategies aimed at solving problems and relating to others in more adaptive way (Samper et al., 2008; Mestre et al., 2012).

In Spain, the percentage of school failure or dropout (i.e., those students who leave the educational system) during the 2012–2013 course was 23.5% (Eurostat, 2014), the double of the European Union percentage (11.9%) for the same period and higher than in other countries, such as the United States and China. Some regions in Spain even reached 29.8% (Veas et al., 2016). The considerable percentage of students that fail school or underachieve can be related, in part, to personal problems, including anxiety, depression, and behavioral problems such as impulsivity and aggression (Broderick and Metz, 2009). Furthermore, school failure is related to alcohol consumption. A study conducted by Goldberg-Looney et al. (2016) found that academic problems explained 5.1% of the variance in adolescents’ alcohol use in a sample of 567 adolescents in Spain.

Impulsivity and risk-taking behavior increase from childhood to adolescence (Oberle et al., 2011; Mestre et al., 2012). The high rate of school failure, along with the increase of emotional disorders, related to stress, anxiety, and aggressive behavior found in Secondary Education and High School students, and even among university students (Amutio and Smith, 2008; Inglés et al., 2014), requires the implementation of psycho-educational programs and emotional self-regulation strategies aimed at activating students’ internal resources, including self-efficacy in order to promote the improvement of interpersonal relationships and academic performance (León, 2008; Amutio et al., 2015a; Gouda et al., 2016), and reducing the risk of school failure. All of the above also implies the need for providing special training to teachers (Kaspereen, 2012; Kemeny et al., 2012; Gouda et al., 2016).

The number of programs to assess and prevent aggressive behavior in adolescence is scarce. In Spain there is a program directed to prevent different types of school violence and aggressiveness, including bullying and cyberbullying (Cyberprogram 2.0; Garaigordobil et al., 2015; Garaigordobil and Martínez-Valderrey, 2016). It consists of different activities to develop coping strategies and other transversal goals, such as developing interpersonal skills (empathy, active listening, social skills, constructive conflict resolution, etc). Additional techniques to reduce aggressiveness include emotional education, improving self-control and problem-solving skills, especially with adolescents showing impulsivity and reactive aggression (Orue et al., 2016).

Another type of intervention, whose effectiveness has been proven are mindfulness techniques. Mindfulness-based interventions have been associated with numerous beneficial outcomes in emotional regulation, including decreased anxiety (Amutio et al., 2015b), depression (Condon et al., 2013), and anger expression reduction (Fix and Fix, 2013; Zenner et al., 2014; Gouda et al., 2016). In the last decade, the practice of mindfulness has proven effective to the development of healthier habits and the generation of better classroom climate (Schonert-Reichl and Lawlor, 2010; López-González et al., 2016), which, in turn, have led to improvements on students’ performance (Franco et al., 2011; Wisner, 2013; López-González and Oriol, 2016). Consequently, a range of mindfulness programs is taking place nowadays in schools as, for example, the *Mindfulness Based Wellness Education (MBWE)* of Toronto University, the Mindfulness in Schools Project (MISP) in England, the *Inner Kids Program, Cultivating Awareness and Resilience in Education* (CARE) and *Stress Management and Relaxation Techniques* (SMART) in the USA. In Spain, it is worth noting the *TREVA Program* (López-González et al., 2016), *Aulas Felices* (Arguís, 2014), and the *Meditación Fluir* Program (Franco et al., 2011).

One definition of mindfulness is to pay attention in a particular way, on purpose, in the present moment, and non-judgmentally (Kabat-Zinn, 2009). Furthermore, mindfulness implies observing the thoughts and emotional reactions that occur at each moment by distancing from them (decentering), that is, not reacting before their presence in the automatic usual way (Krishnakumar and Robinson, 2015; Peters et al., 2015), thus, breaking the thinking-feeling-acting typical pattern. Thereby, and through continuous practice, students learn to concentrate on the task they are performing, without allowing their minds to digress or get distracted. This provides students with a new perspective that facilitates reflection and learning.

Currently, extensive data support the use of mindfulness in the achievement of greater levels of relaxation, well-being, and improvement of academic performance (Beauchemin et al., 2008; León, 2008; Schonert-Reichl and Lawlor, 2010; Franco et al., 2011; Choi et al., 2012; Amutio et al., 2015c). In addition, recent neurodevelopmental findings show that mindfulness and social-emotional learning programs implemented in regular school curricula improve executive functions in children and adolescents in terms of inhibitory control, enabling them to manage excessive levels of negative emotions that interfere with academic performance (Davidson et al., 2012; Sanger and Dorjee, 2015). Studies conducted on both clinical and non-clinical samples (Zenner et al., 2014) include children and adolescents with attention deficit hyperactivity disorder (van de Weijer-Bergsma et al., 2012; Van der Oord et al., 2012; Cardoso-Moreno et al., 2015), anxiety (Beauchemin et al., 2008), hostility (Sibinga et al., 2011), and externalizing disorders, such as impulsivity (Bögels et al., 2008), as well as adolescents at risk (Bluth et al., 2016).

In spite of these findings, meditation treatment effects among youth are relatively unknown (Black et al., 2009). Currently, controlled studies measuring the impact of mindfulness training on reducing impulsivity and hostility levels of adolescents in the classroom barely exist. Moreover, little is known about the association of mindfulness with decreased emotional reactivity and improved impulse-control, especially in adolescents. Among the few studies conducted, we highlight those of Oberle et al. (2011) and Fishbein et al. (2016). These studies consisted of an intervention in adolescents with high-risk behaviors and are among the first ones assessing the effectiveness of a mindfulness-training program to reduce impulsivity and aggression levels of adolescents in the classroom.

Given the current situation, the aim of this study is to prove the effect of a mindfulness training psycho-educational program applied to a group of adolescents with behavioral problems in the classroom on their impulsivity and aggression levels, assuming the following hypothesis:

H1: Those adolescents with behavioral problems in the classroom and participating in a mindfulness training psycho-educational program will experience a significant decrease in their impulsivity levels compared to the group of adolescents with behavioral problems in the classroom that were not part of the intervention program.
H2: Those adolescents with behavioral problems in the classroom and participating in a mindfulness training psycho-educational program will experience a significant decrease in their aggression levels compared to the group of adolescents with behavioral problems in the classroom that were not part of the intervention program.

## Materials and Methods

### Participants

Twenty seven students with ages from 12 to 19 years (Mean = 15.85; Standard deviation = 2.38), who were attending a public high school center located in the province of Granada participated in this study. In this sample, 59% of the participants were boys and 41% girls. The control group was made up of 14 individuals (57% boys and 43% girls), while the 13 individuals remaining were sent to the experimental group (62% boys and 38% girls).

### Instruments

#### Barratt Impulsivity Scale (BIS-11) (Patton et al., 1995)

This questionnaire is composed of 30 items grouped in three impulsivity sub-scales:

– Cognitive impulsivity (8 items): tendency to make quick decisions.
– Motor impulsivity (10 items): propensity to act solely on the spur of the moment, without thinking of the consequences.
– Non-planned impulsivity (12 items): indicates lack of planning of future actions.

Each item comprises four Likert-type answer options: rarely/never, occasionally, often, and almost always/always. The score of each sub-scale is calculated by adding up the partial scores obtained in each item. The total score is the sum of all the items.

The Spanish version of this scale, created by Oquendo et al. (2001) was administered. The internal consistency of the different scales used in the study sample was obtained using Cronbach’s alpha, which presented values ranging from 0.77 to 0.92.

#### Aggression Questionnaire (AQ) (Buss and Perry, 1992)

This instrument is used to measure aggressiveness. In this study, the Spanish version created by Rodríguez et al. (2002) was used. The questionnaire is composed of 29 items with five Likert-type answer options (1 = *very few times*, 5 = *lots of times*) that form the following scales:

– Physical aggressiveness (9 items): it refers to physical behaviors that hurt or harm other people.
– Verbal aggressiveness (5 items): it is related to verbal behaviors that hurt or harm other people.
– Hostility (8 items): assesses the cognitive aspects of aggression.
– Anger (7 items): assesses the emotional and affective aspects of aggression.

Regarding the reliability coefficients for the studied sample, these range from 0.72 in the verbal aggression scale to 0.85 in the physical aggression scale.

### Procedure

Firstly, to obtain the sample, an interview with the principal, the head of studies and the head of the counseling department of the high school center was conducted in order to explain the study objectives and to ask permission and collaboration for the application of the questionnaires. Subsequently, 27 students that had been sent more than five times to the counseling room during the first term of the school year due to misbehavior in the classroom were selected as the sample. All parents provided informed consent. The study was approved by the Committee of Bioethics of the University of Almería, Spain. The registered data for each of the instruments was alphanumerically coded, ensuring confidentiality and anonymity, in order to comply with the Personal Data Protection Act by the Ethics Committee for Research related to Human Beings (CEISH). International ethical guidelines for studies with human subjects described in the Nuremberg Code and in the Declaration of Helsinki were applied (Kim, 2012).

Students were randomly assigned to the control (*n* = 14) and experimental groups (*n* = 13), controlling for sex and grade to avoid the interference of these variables in the results. Once the sample was obtained, pre-test measurements for the different dimensions of the impulsivity and aggressiveness variables were obtained by asking the participants to individually complete the questionnaires.

Subsequently, the intervention program was applied to the experimental group over 10 weekly sessions that took place during the counseling hours of the students. This intervention program consisted in the learning and daily practice of a mindfulness technique named *Meditación Fluir* for 15 min (Franco et al., 2011, 2014). The principal goal of this practice is attempting neither to control thoughts, sensations or feeling nor altering or change them by new ones, but the contrary, i.e., let them free to come and go, and accepting any personal sensation and feeling that may arise spontaneously.

Therefore, the essence of this technique is being aware of what happens in the mind and body in a passive way, without exerting any effort on modifying or changing the situation, perceiving things as they truly are and as they occur in every moment. Thus, this mindfulness practice enables the capacity to observe thoughts and other mental activity without getting involved in it—i.e., without analyzing, judging, or evaluating it—, breaking people’s habit of being carried away and dragged by automatic and uncontrolled thoughts. In other words, students become aware of the presence of thoughts during the practice, but do not reflect upon their content or truthfulness, realizing that thoughts and sensations change at every moment and are constantly flowing. Consequently, through this technique, students understand from experience that thoughts continuously appear and disappear in a constant flow. In this way, students learn to be present, open and balanced against any mental or emotional phenomenon or process.

Another aspect of the mindfulness program that was learned and put into practice during the 10 sessions was the performance of *body-scan* exercises (Kabat-Zinn, 2009). Body-scan is a technique in which attention is orderly and systematically paid to different body parts, subsequently expanding consciousness to the whole body, thereby achieving holistic awareness of the body without making value judgments nor trying to change or eliminate anything (e.g., proprioceptive and interoceptive sensations, mental reactions, etc.), that is, being always present.

Once mindfulness training was completed in the experimental group, post-test measurements for the different dimensions of the impulsivity and aggressiveness variables were obtained by the same method used in the pre-test stage. Once the study, was completed the mindfulness-based training program was delivered to the control group.

### Design and Data Analysis

A quasi-experimental comparison group pre-test–post-test design with experimental and control groups was used to analyze the effects of the mindfulness training program (independent variable) on the different dimensions of the impulsivity and aggressiveness variables (dependent variables).

The existence of statistically significant differences between the mean scores of the control and experimental group in the different dimensions of impulsivity and aggression in each stage of the study was proved by means of the non-parametric statistical test *Mann–Whitney U* for independent samples, since data did not adjust to the normal probability distribution.

Next, non-parametric statistical test *Wilcoxon* for comparing related samples was used in order to prove the existence of statistically significant differences between the mean scores of the different dimensions of impulsivity and aggression in each stage of the study, for both the control and experimental groups.

Finally, Cohen’s *d* and the percentage of change in the pre-test–post-test scores were used to determine the magnitude of the change experienced after the intervention program in the different impulsivity and aggression dimensions in the experimental group. All the statistical analyses were computed using the SPSS 22.0 package.

## Results

Firstly, variable means and standard deviations corresponding to the control and experimental group in each stage of the study were calculated (**Table 1**).

**Table 1:** Pre-test and post-test means and standard deviations corresponding to the different impulsivity and aggressiveness dimensions for the control and experimental groups.

	Pre-test				Post-test			
	Control		Experimental		Control		Experimental	
Variable	*M*	*SD*	*M*	*SD*	*M*	*SD*	*M*	*SD*
**Impulsivity**								
Cognitive	22.98	4.84	23.48	4.79	23.45	4.28	18.93	3.95
Motor	28.81	5.17	27.94	4.93	27.53	5.34	24.36	4.56
Non-planning	32.43	6.93	31.07	6.44	31.91	5.52	27.91	5.87
Total	84.22	11.04	82.49	10.27	82.89	11.04	71.2	9.16
**Aggressiveness**								
Physical	27.21	5.28	28.07	5.49	26.87	4.51	24.17	4.14
Verbal	15.45	3.75	16.04	4.11	16.09	4.68	12.12	3.84
Hostility	25.18	5.30	23.95	4.88	26.36	3.98	19.36	4.01
Anger	23.04	5.53	23.89	6.04	24.11	6.87	20.03	5.31

*Mann-Whitney U test* for independent samples on the pre-test scores revealed no statistically significant differences between the control and experimental pre-test mean scores in the study variables. Contrarily, statistically significant differences did appear between the control and experimental groups at post-test in all the dimensions of impulsivity and aggressiveness (**Table 2**).

**Table 2:** Mann-Whitney U test for independent samples of the pre-test and post-test differences between the control and experimental groups for the different dimensions of impulsivity and aggressiveness.

	Pre-test		Post-test	
Variable	*z*	*p*	*z*	*p*
**Impulsivity**				
Cognitive	0.345	0.733	3.81	0.006^∗∗^
Motor	0.423	0.637	2.38	0.031^∗^
Non-planned	0.516	0.475	3.31	0.014^∗^
Total	0.741	0.309	3.68	0.008^∗∗^
**Aggressiveness**				
Physical	0.629	0.543	3.16	0.019^∗^
Verbal	1.02	0.248	2.74	0.026^∗^
Hostility	693	0.494	3.39	0.013^∗^
Anger	0.759	0.328	3.94	0.005^∗∗∗^

*^∗∗∗^*p* = 0.005; ^∗∗^*p* < 0.01; ^∗^*p* < 0.05.*

After conducting *Wilcoxon* test for related samples on the experimental group scores, statistically significant differences were observed when comparing the pre-test and post-test scores in all the dimensions of impulsivity and aggressiveness. No significant differences were found in such variables after the pre-test and post-test comparisons for the control group (**Table 3**).

**Table 3:** Wilcoxon test for related samples of the pre-test and post-test differences between the control and experimental groups for the different dimensions of impulsivity and aggressiveness.

	Control		Experimental	
Variable	*z*	*p*	*z*	*p*
**Impulsivity**				
Cognitive	0.928	0.375	-4.36	0.002^∗∗∗^
Motor	-0.829	0.244	-2.29	0.038^∗^
Non-planned	-0.468	0.614	-3.17	0.019^∗^
Total	-0.679	0.438	-3.43	0.012^∗^
**Aggressiveness**				
Physical	-0.619	0.456	-3.97	0.005^∗∗^
Verbal	0.731	0.314	-2.46	0.033^∗^
Hostility	0.132	0.842	-3.16	0.019^∗^
Anger	0.607	0.557	-4.44	0.003^∗∗^

*^∗∗∗^*p* < 0.005; ^∗∗^*p* = 0.005; ^∗^*p* < 0.05.*

With the purpose of assessing the magnitude of the change occurred in the experimental group between pre-test and post-test scores, Cohen’s *d* (1988) was used, with values above 1.5, between 1.5 and 1, and between 1 and 0.5 indicating very important, important and medium changes, respectively. Cohen’s *d* showed the existence of very important changes in the cognitive impulsivity, total impulsivity and hostility dimensions, and medium to high changes in the other dimensions of impulsivity and aggressiveness (**Table 4**).

**Table 4:** Cohen’s *d* and pretest-post-test percentage change in the experimental group for the different dimensions of impulsivity and aggressiveness.

Variable	*d* Pre-Post	*%* Pre-Post
**Impulsivity**		
Cognitive	1.04	-19.38
Motor	0.753	-12.81
Non-planned	0.514	-10.17
Total	1.16	-13.69
**Aggressiveness**		
Physical	0.803	-13.91
Verbal	0.995	-24.44
Hostility	1.03	-19.17
Anger	0.679	-16.16

Finally, the percentage of change between pre-test and post-test scores in the experimental groups for the different impulsivity and aggressiveness dimensions was calculated. **Table 4** shows reductions of approximately from 24% in the verbal aggression dimension and to around 10% in the non-planned impulsivity dimension.

## Discussion

As a result of the application of the *Fluir meditation technique* (Franco et al., 2011) during 10 weeks, significant reductions in all the dimensions of impulsivity and aggressiveness levels occurred in the experimental group composed of high school students, thus, confirming the hypotheses. The obtained results are in line with the findings of other studies (Oberle et al., 2011; Fishbein et al., 2016). There may be different explanations for the obtained results, namely decrease in rumination (Borders et al., 2010; Peters et al., 2015; Orue et al., 2016), reduction in hostile affect, including frustration and anger feelings (Kemeny et al., 2012; Krishnakumar and Robinson, 2015) and increase in self-control before stressors (Broderick and Metz, 2009; Yusainy and Lawrence, 2014), amongst others. In this way, the capacity to regulate attention and emotion are forms of self-regulation that support dispositions conducive to learning and maintaining positive social relationships (Flook et al., 2015).

Aggressiveness is a complex phenomenon involving multiple factors, including psychosocial. Aggressive behavior is seen by some adolescents as a strategy to avoid future victimization or rejection, while for others it is interpreted as an opportunity to achieve the desired popularity among peers (Estévez et al., 2014). Our data confirm the efficacy of the *Meditación Fluir-mindfulness technique.* However, integrative psycho-educational intervention programs oriented to promote emotional education and in values within the school centers (Peña and Canga, 2009; Sanger and Dorjee, 2015) are needed in order to reduce the high rates of violent behavior in adolescence, improve classroom climate and diminish the risk of school failure. The development of the ability to delay gratification, along with an adequate emotional self-regulation that includes rumination control and a negative assessment of the consequences of using aggression, would be key elements to be addressed by psycho-educational programs directed to adolescent population in school and community environments.

The practice of mindfulness can help students focus on the present, thus, reducing obsessive ruminations and enhancing the experience of positive emotions, as well as diminishing the probability of involvement in impulsive behaviors, which, in general, tend to aggravate the same emotional problems that want to be alleviated or solved by the use of aggression (Fix and Fix, 2013; Amutio et al., 2015a). In turn, this training allows recognizing the first signs of aggressive impulses in such a way that they are more likely to be inhibited by using the skills developed through the practice of mindfulness (i.e., acceptance and equanimity). Additionally, mindfulness has also been linked to increased compassion for both the self and others, which may foster a great sense of interconnectedness and facilitate the response to potential conflict with non-aggressive approaches (Condon et al., 2013).

There are hardly any studies in Spain on the influence of mindfulness in aggression or impulsivity in adolescents, since a majority are focused on stress or anxiety and are mainly directed to the adult population. Although research on mindfulness, especially with children and adolescents, is still in relatively early stages, an increasing number of studies have shown the potential benefits of mindfulness practices to students’ physical health and psychosocial well-being, enhancing academic performance and diminishing the risk of school failure (Franco et al., 2011, 2014; Amutio et al., 2015a; López-González et al., 2016). This is a very important concern given that school failure in adolescence is a strong predictor of other high-risk behaviors, including delinquency, substance abuse and adolescent pregnancy (Stratton, 2006; Morley et al., 2016). More generally, a negative attitude toward school is, in fact, a risk factor for aggressive behaviors (Estévez et al., 2014). Conversely, school connectedness, engagement, academic achievement, and academic enjoyment may protect against alcohol use, suspension, lower educational aspirations, and poor academic performance (Goldberg-Looney et al., 2016).

## Conclusion

The main limitation of this study is the reduced size of the sample. Further studies need to be carried out with bigger samples in order to determine specific causal mechanisms (e.g., changes in prefrontal brain structures, decrease in ruminations, emotional self-regulation, development of compassion) of the observed effects. However, this is one of the first studies showing the effectiveness of a mindfulness program for reducing impulsive and aggressive behaviors in the classroom. In an era of budget cuts, this type of group psycho-educational programs implies a considerable optimization of the economic and social resources invested on education in order to improve academic performance and decrease school failure.

## Author Contributions

CF: Data collection and analyses. AA: Data interpretation, introduction, and discussion. LLG: Bibliography, literature review, and corrections. XO: Literature review and procedure. CMT: Literature review and article revision.

## Conflict of Interest Statement

The authors declare that the research was conducted in the absence of any commercial or financial relationships that could be construed as a potential conflict of interest.
